# The impact of informal care provision on the quality of life of adults caring for persons with mental health problems: A cross-sectional assessment of caregiver quality of life

**DOI:** 10.1177/20551029241262883

**Published:** 2024-08-06

**Authors:** Leonarda G M Bremmers, Isabelle N Fabbricotti, Eleonora S Gräler, Carin A Uyl-de Groot, Leona Hakkaart-van Roijen

**Affiliations:** 6984 Erasmus University Rotterdam, The Netherlands

**Keywords:** caregivers, cross-sectional studies, deinstitutionalization, quality of life, mental disorders

## Abstract

This study assessed the quality of life of informal caregivers for individuals with mental health problems in the Netherlands. An online survey was administered to a panel of informal caregivers in June 2020 (*n* = 261). Informal caregivers of persons with mental problems were found to have an exceptionally low quality of life. A high subjective burden (*p* < .001), lower perseverance time (*p* = .016), low caregiver overall health status (*p* = .004) and psychological wellbeing (*p* = .008), younger caregiver age (*p* = .011), child caregiving responsibilities (*p* = .025), and no social support network (*p* = .038) were associated with worse quality of life scores. These findings shed light on the significant challenges faced by informal caregivers of persons with mental health problem. This raises concerns about the long-term sustainability of informal care and mental healthcare reform.

## Introduction

The profound impact of mental health disorders is increasingly recognized, shedding light on the daily challenges faced by patients and their informal caregivers. Informal caregivers, often family members or close friends, play a pivotal role in providing long-term support, understanding, and care to mental health patients (Van Ginneken and Kroneman, 2015). The wellbeing of these caregivers is an integral aspect of the broader societal fabric, as their experiences can significantly influence the overall quality of life for both themselves and the individuals under their care (Phillips et al., 2023; Rodriguez et al., 2019). Informal caregivers for persons with mental health problems often characterize their caregiving experience as burdensome-reporting that their “emotional or physical health, social life, or financial status had suffered as a result of caring for their relative” (Zarit et al., 1986: p. 261). The impact of caregiving is well-established for burden-related outcomes in this population (Bremmers et al., 2022). However, we currently lack a sufficient research base that considers comprehensive outcomes, such as quality of life. Given the complexity of the caregiving experience, comprehensive outcomes should be considered since caregiver quality of life reflects the positive and negative effects of the caregiving situation from the informal caregiver’s perspective (Martin et al., 2021).

Providing informal care to a person with mental health problems presents specific challenges. Mental health disorders often require ongoing and long-term care and can have a significant impact on a patient’s ability to communicate, interact with others, and perform daily tasks. Informal caregivers may also face difficulties in understanding and managing the individual’s symptoms. This can be emotionally taxing for the informal caregiver, who may struggle with feelings of stress, burnout, and isolation (Denee et al., 2023). Moreover, informal caregivers often lack access to caregiver support and resources, which can further exacerbate their caregiving burden (Latifian et al., 2022; Milačić-Vidojević et al., 2014). Informal caregivers are also often excluded from their care recipients’ treatment process (Seif et al., 2022) and subjected to courtesy stigma, which includes blaming caregivers for the onset of the mental health disorder and others assuming the caregiver lacks competence in their caregiving role (Angermeyer et al., 2003). Consequently, informal caregivers for persons with mental health problems constitute a particularly vulnerable group of caregivers (de Boer et al., 2019) that report worse quality of life outcomes when compared to other caregiving populations (Gupta et al., 2015; Hastrup et al., 2011).

Existing quality of life literature has demonstrated that informal caregivers report a low quality of life across a variety of mental health disorders, including schizophrenia (Boyer et al., 2012; Cohen et al., 2022; Hsiao et al., 2020; Khatimah et al., 2022; Margetic et al., 2013; Meng et al., 2021; Yerriah et al., 2022), autism spectrum disorder (Rattaz et al., 2017; Sonido et al., 2022), depressive disorders (Zendjidjian et al., 2012), and bipolar disorder (Cohen et al., 2022). These studies generally focus on a specific disorder, most frequently schizophrenia, thus limiting their generalizability. When conducting quality of life research on informal caregivers for mental health disorders in general, as opposed to solely focusing on a specific disorder, it allows for a broader exploration of common themes, challenges, and support needs that may be applicable across various mental health conditions. Furthermore, the existing empirical evidence has largely been conducted outside of Europe. In the Netherlands, the caregiving culture includes long-term care and support from the formalized healthcare system, with a strong emphasis on familial support. There is a societal expectation that family members engage in caregiving roles, ensuring that patients can remain at home for as long as possible (Van Ginneken and Kroneman, 2015). The cultural context, societal norms, and the level of support provided by the country’s healthcare and social systems all play crucial roles in shaping the experiences of caregivers (Broese van Groenou and de Boer, 2016; Verbakel, 2018). Hence, there is a need for further research on quality of life within the Dutch caregiving context, including its respective determinants.

Quality of life determinants have been broadly conceptualized into three categories: the caregiving situation, caregiver factors, and environmental factors (White et al., 2004; Wong et al., 2012). The caregiving situation encompasses factors related to the characteristics of the care recipient’s illness and the strains associated with caregiving, such as objective and subjective burden. Caregiver factors are defined as the characteristics of the informal caregivers, including sociodemographic characteristics and personal attributes, whilst environmental factors relate to external support that the informal caregiver may receive (Wong et al., 2012). A variety of aspects related to the caregiving situation, caregiver factors, and environmental factors have been identified as quality of life determinants in previous empirical literature (Boyer et al., 2012; Cheng et al., 2022; Cohen et al., 2022; Hsiao et al., 2020; Khatimah et al., 2022; Leng et al., 2019; Margetic et al., 2013; McDaid and Park, 2022; Meng et al., 2021; Rattaz et al., 2017; Wong et al., 2012). However, the identified determinants vary across studies due to unrepresentative sample sizes and differing study contexts, such as country, caregiving culture, and mental health disorder of care recipient (e.g., Boyer et al., 2012; Cheng et al., 2022; Cohen et al., 2022; Hsiao et al., 2020; Khatimah et al., 2022; Leng et al., 2019; Margetic et al., 2013; McDaid and Park, 2022; Meng et al., 2021; Rattaz et al., 2017; Wong et al., 2012). Furthermore, the role of well-recognized quality of life determinants, such as gender (Sharma et al., 2016), are not well-understood in this caregiving population and warrant further research. Studying the determinants of quality of life in informal caregivers is crucial for developing targeted interventions, informing policies, and ultimately improving the well-being of caregivers and the quality of care they provide to their loved ones. Hence, this study aims to assess the quality of life of informal caregivers for persons with mental health problems and investigate the respective determinants.

## Research methods

This cross-sectional study was reported in accordance with the STROBE Checklist guidelines. Refer to the appendix for the completed STROBE checklist (Vandenbrouckel et al., 2007).

### Study design and participants

A cross-sectional data analysis was conducted using data from an online survey administered to a panel of Dutch informal caregivers (*n* = 1006) in June 2020, 3 months after the start of the COVID-19 lockdown in the Netherlands (Gräler et al., 2022).

Respondents were considered eligible for inclusion if they reported that they were adults (≥18 years of age) that provided a minimum of 2 h of informal care and support per week to a person (≥18 years of age) with a reported psychological disorder (e.g., depression or anxiety disorders) or psychosocial problem (e.g., addiction) for at least a 3-month period. For the final analysis, 261 respondents were included. A total of 734 respondents were excluded because they did not comply with the inclusion criteria (e.g., care recipient had a somatic disorder). A further eleven respondents were excluded due to inaccurate responses. There were no missings, due to the use of forced-choice questions in the survey.

The study protocol was reviewed and approved by the Research Ethics Review Committee of the Erasmus School of Health Policy & Management (reference: IRB 20–16). All respondents provided informed consent prior to completing the survey and were free to stop participation at any moment in time.

### Setting

Data collection occurred during the first wave of the COVID-19 pandemic. The onset of the COVID-19 pandemic is characterized by a rapidly evolving healthcare landscape, with mental health services being amongst the most severely disrupted (World Health Organization, 2020). This included the (partial) suspension and closure of health and social support services (De Boer et al., 2020a) and rapid implementation of telemedicine (Chow et al., 2021; Feijt et al., 2020). These disruptions have been evidenced to result in differences in the caregiving situation and environmental factors, such as limited access to healthcare services and social networks for support (De Boer et al., 2020a; EuroCarers and IRCCS-INRCA, 2021; Pan et al., 2021; Raiber et al., 2022). Additionally, the COVID-19 pandemic and the respective containment measures have been evidenced to affect the intensity of caregiving, with some informal caregivers reporting an increase in intensity due a delay in moving to a care facility or loss of support, while in other instances, the measures have hindered the provision of informal care (De Boer et al., 2020a). Quality of life research conducted during this period has been limited to general caregiving populations. Their findings highlighted that informal caregivers’ quality of life suffered during this time, with some populations being more affected than others. This included women, caregivers who reported being unemployed and having a younger age, a lower socioeconomic status, and having child caregiving responsibilities (Gräler et al., 2022; Zwar et al., 2023). To the best of our knowledge, no quality of life research has been conducted in informal caregivers of persons with mental health problems during this time period.

### Measurements

The survey collected data on the caregiving situation, caregiver factors, environmental factors, COVID-19 related concerns, and the caregiver’s quality of life.

#### Caregiver quality of life determinants

Determinants were selected based on their theoretical relevance and use in previous peer-reviewed literature. Determinants were then categorized using the Quality of Life model, a framework for investigating how the caregiving experience impacts an informal caregiver’s quality of life (White et al., 2004; Wong et al., 2012).

#### Caregiving situation

Caregiving situation variables were subjective burden (score), kinship with care recipient (0 “not a family member, including friends, acquaintance or neighbour” 1 “family member, including partner, mother(-in-law), father(-in-law), daughter, son, sister, brother or other family member”), lives with caregiver (0 “does not live with the caregiver” 1 “lives with caregiver”), psychological wellbeing of care recipient (score), intensity of caregiving (hours per week), duration of caregiving (months), supervision of care recipient (0 “care recipient cannot easily stay alone for a couple of hours” 1 “care recipient cannot easily stay alone”), perseverance time (0 “less than 2 years” 1 “two or more years”), and care recipient co-morbidity status (0 “does not have any comorbidities” 1 “has at least 1 comorbidity”).

Subjective burden was assessed using the self-rated burden scale (SRB), which employs a horizontal visual analogue scale (VAS) to judge the burden of caregiving on a scale from 0 (“not straining at all”) to 10 (“much too straining;” van Exel et al., 2004). The psychological wellbeing of the care recipient was determined with a horizontal VAS scale from 0 (“worst imaginable psychological wellbeing”) to 10 (“best imaginable psychological wellbeing;” Hoefman et al., 2013). Intensity of caregiving was measured using an adapted version of the intensity of informal care questionnaire, which assessed the hours of support with household tasks, support with self-care tasks, emotional support, and practical support (Hoefman et al., 2013). Perseverance time is a validated single-question instrument that asks informal caregivers to report “if the informal care situation stays as it is now, how long will you be able to cope with the care?” (Kraijo et al., 2014; Richters et al., 2016). For the purpose of this study, the response categories were dichotomized for the perseverance time variable.

#### Caregiver factors

The following caregiver factors were measured: caregiver gender (0 “male” 1 “female”), highest level of attained education (base: lower level, including primary education or practical secondary education; X1: middle level, including other secondary education or practical tertiary education; X2: higher level, including other levels of tertiary and further education), employment status (0 “unemployed, including retirement, student, homemaker” 1 “employed, including full- and part-time employment”), caregiver age (years), ability to make ends meet (0 “easy to make ends meet” 1 “difficult to make ends meet”), and child caregiving responsibilities (0 “does not care for a child under 18 years of age” 1 “provides care to a child under 18 years of age”).

Caregiver overall health status was determined using the EQ-5D-5L, a VAS which records the respondent’s self-rated health on a horizontal scale ranging from 0 (“the worst health you can imagine”) and 10 (“the best health you can imagine;” Herdman et al., 2011). Caregiver psychological wellbeing was determined using the MHQoL-VAS. The MHQoL-VAS assesses the respondent’s self-rated overall psychological wellbeing on a horizontal scale from 0 (“worst imaginable psychological wellbeing”) to 10 (“best imaginable psychological wellbeing;” van Krugten et al., 2022).

#### Environmental factors

The selected environmental factor was social support network, with 0 coded as “does not receive social support” and 1 “receives social support.”

#### Control variable

COVID-19 related concerns were employed as a control variable, with 0 coded as “no COVID-19 related concerns” and 1 as “COVID-19 related concerns.”

#### Quality of life measure

Caregiver quality of life was measured with the CarerQol. The Dutch-language CarerQoL combines a subjective burden measure that provides a comprehensive description of the caregiving situation (CarerQoL-7D) with a valuation of informal care in terms of happiness (CarerQoL-VAS). The questionnaire operationalizes the impact of informal care into five negative dimensions (relational problems, mental health problems, problems combining daily activities with care, financial problems, and physical health problems) and two positive dimensions (fulfilment from caregiving and support with lending care). Three response options are employed for questions: “no,” “some,” and “a lot.” For the CarerQoL-VAS, higher scores indicate a happier informal caregiver, with 0 being “completely unhappy” and 10 “completely happy” (Brouwer et al., 2006). A weighted sum score of the CarerQoL was employed, which was calculated based on the preferences of the Dutch general adult population. Higher scores indicate a better care-related quality of life (Hoefman et al., 2014).

### Statistical analyses

Statistical analyses were conducted in IBM SPSS Statistics (version 28) with a statistical significance (α) of 0.05. The complete syntax is available upon request.

Descriptive statistics (mean, standard deviation (SD), frequency distributions) were computed to report the caregiving situation, caregiver factors, and environmental factors.

#### Quality of life assessment

Descriptive statistics (mean, SD, frequency distributions) were calculated for the care-related quality of life.

#### Quality of life determinants

A multiple linear regression analysis was conducted for the dependent variable, care-related quality of life. The caregiver quality of life determinants and the control variable, COVID-19 related concerns, were included in the model.

## Results

### Characteristics of respondents

A total of 261 respondents were included in the final analyses (see Table 1). In terms of caregiving situation, informal caregivers report an average subjective burden of 5.7 (scale 0–10; SD = 2.4) and a caregiving intensity of 29.7 h per week (SD = 29.7). The duration of caregiving ranged from 3 to 576 months (average = 94.7 months; SD = 92.9 months). A total of 85 care recipients (32.6%) did not have comorbidity. Most care recipients did not live with their informal caregiver (*n* = 192; 73.6%), can easily stay alone for a couple of hours (*n* = 182; 69.7%), had a perseverance time of less than 2 years (*n* = 131; 50.2%) and were a family member (*n* = 215; 82.4%). The average psychological wellbeing score of care recipients was 5.1 (SD = 2.0). For caregiver factors, a majority of informal caregivers were female (*n* = 162; 62.1%). Care recipient age averaged at 47.1 years (SD = 15.9). A total of 117 informal caregivers (44.8%) had completed a higher level of education and 102 were unemployed (39.1%). Additionally, informal caregivers reported having difficulties making ends meet (*n* = 146; 55.9%) and provided care to a child under 18 years of age (*n* = 81; 31.0%). Concerning relevant environmental factors, a total of 204 (78.2%) informal caregivers reported receiving some form of social support.

**Table 1.** Characteristics of informal caregivers and their caregiving situation (*n* = 261).

Caregiving situation	
Subjective burden *mean score (min.-max.; SD)*	5.7 (0-10; 2.4)
Kinship with care recipient *n (%)*	
Not a family member, including friends, acquaintances, or neighbours	46 (17.6)
Lives with care recipient *n (%)*	
Does not live with caregiver	192 (73.6)
Psychological wellbeing of care recipient *mean score (min.-max.; SD)*	5.1 (0-10; 2.0)
Intensity of caregiving *mean hours per week (min.-max.; SD)*	29.7 (2-160; 29.7)
Duration of caregiving *mean months (min.-max.; SD)*	94.7 (3-576; 92.9)
Supervision of care recipient *n (%)*	
Care recipient cannot easily stay alone	79 (30.3)
Perseverance time *n (%)*	
Two or more years	130 (49.8)
Care recipient comorbidity status *n (%)*	
No, does not have a comorbidity	85 (32.6)
Caregiver factors	
Caregiver gender *n (%)*	
Male	99 (37.9)
Highest attained education level *n (%)*	
Lower level of education	33 (12.6)
Middle level of education	111 (42.5)
Higher level of education	117 (44.8)
Employment status *n (%)*	
Unemployment, including retirement, student, and homemaker	102 (39.1)
Caregiver age *mean score (min.-max.; SD)*	47.1 (19-84; 15.9)
Ability to make ends meet *n (%)*	
Difficult to make ends meet	146 (55.9)
Child caregiving responsibilities *n (%)*	
Provides care to a child under 18 years of age	81 (31.0)
Environmental factors	
Social support network *n (%)*	
No social support	57 (21.8)
Control variable	
COVID-19 related concerns *n (%)*	
No COVID-19 concerns	37 (14.2)

*Notes*. max. maximum; min. minimum; SD standard deviation, n number.

### Quality of life assessment

**Figure 1.**
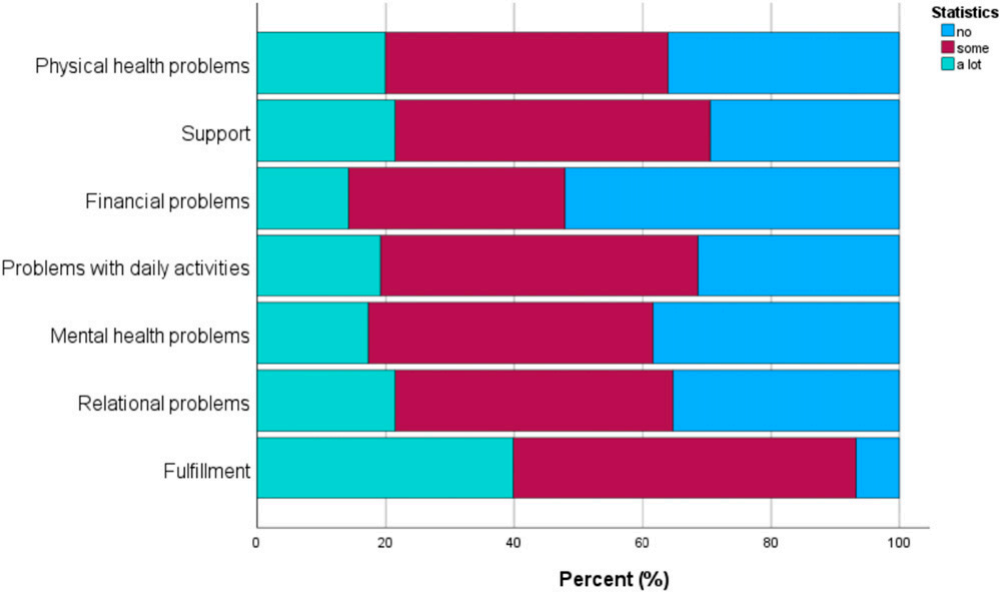
Percentage of informal caregivers experiencing physical health problems, support, financial problems, problems with daily activities, mental health problems, relational problems, and fulfilment (%).

Informal caregivers reported a low care-related quality of life (mean = 69.8, SD = 19.64). Almost all informal caregivers reported fulfilment from caregiving, and the majority received some support with their care tasks (refer to Figure 1). Most informal caregivers had at least some relational problems with their care recipient or problems with combining their caregiving tasks with other daily activities. Almost two-thirds reported having at least some physical and/or mental health problems. Approximately half of the informal caregivers indicated that they had at least some financial problems due to their caregiving.

### Quality of life determinants

The multiple linear regression was statistically significant (R^2^ = 0.463, F(20, 240) = 10.365, *p* < .001). It was found that subjective burden (β = −0.294, *p* < .001), perseverance time (β = 0.155, *p* = .016), caregiver overall health status (β = 0.192, *p* = .004), caregiver psychological wellbeing (β = 0.172, *p* = .008), caregiver age (β = 0.164, *p* = .011), child caregiving responsibilities (β = −0.127, *p* = .025), and social support network (β = 0.113, *p* = .038) were significantly associated with care-related quality of life. Hence, a high subjective burden, lower perseverance time, low caregiver overall health status and psychological wellbeing, younger caregiver age, having child caregiving responsibilities, and no social support network were associated with a lower quality of life. Refer to Table 2 for the complete regression model.

**Table 2.** Determinants for care-related quality of life.

	Standardized Coefficients		
	β^a^	t	*p*-value
Caregiving situation			
Subjective burden	**−0.294**	**−5.206**	**<0.001**
Kinship with care recipient	−0.071	−1.384	0.168
Lives with care recipient	0.046	0.798	0.425
Psychological wellbeing of care recipient	−0.028	−0.525	0.600
Intensity of caregiving	−0.041	−0.730	0.466
Duration of caregiving	−0.108	−1.941	0.053
Supervision of care recipient	−0.084	−1.567	0.118
Perseverance time	**0.155**	**2.436**	**0.016**
Care recipient comorbidity status	0.058	1.112	0.267
Caregiver factors			
Caregiver overall health status	**0.192**	**2.868**	**0.004**
Caregiver psychological wellbeing	**0.172**	**2.660**	**0.008**
Caregiver gender	0.031	0.629	0.530
Highest attained education level (base: lower level, including LO, LBO and MAO)			
*Middle level, including MBO and HAO*	0.115	1.470	0.143
*Higher level, including WO and HBO*	0.116	1.434	0.153
Employment status	0.093	1.678	0.095
Caregiver age	**0.164**	**2.568**	**0.011**
Ability to make ends meet	−0.005	−0.087	0.931
Child caregiving responsibilities	**−0.127**	**−2.259**	**0.025**
Environmental factors			
Social support network	**0.113**	**2.084**	**0.038**
Control variable			
COVID-19 related concerns	0.011	0.211	0.833
Model summary	R^2^ = 0.463		
	F(20, 240) = 10.365, *p* < .001		

*Notes*. Bold values: *p* < .05.

^a^β: standardized beta coefficient (represents the change of the standard deviation in quality of life score resulting from a change of one standard deviation in the independent variable).

## Discussion

The findings of this research shed light on the significant challenges faced by informal caregivers of persons with mental health problems, revealing an exceptionally low quality of life within this population. This overall low score indicates that this group of informal caregivers is generally dissatisfied with their wellbeing and that their satisfaction with various aspects of their life is significantly diminished. Furthermore, this research highlights the intricate interplay of determinants influencing the quality of life for informal caregivers of persons with mental health problems. We identified various non-modifiable and modifiable factors associated with a diminished caregiver quality of life, including elevated subjective burden, reduced perseverance time, compromised caregiver overall health status and psychological well-being, younger caregiver age, increased child caregiving responsibilities, and absence of a social support network. The discussion will delve into the implications of our findings and the various determinants identified in the study that contribute to the observed low quality of life, providing insights into the multifaceted nature of caregiver experiences.

The low caregiver quality of life reported by informal caregivers of individuals with mental health problems in our study population, align with and reinforce prior quality of life studies. The pervasive dissatisfaction with overall well-being observed in our study mirrors trends reported in comparable research, indicating a widespread struggle among this caregiving group that is not limited to a certain geographic location (Cheng et al., 2022; Hastrup et al., 2011; Leng et al., 2019; McDaid and Park, 2022; Wong et al., 2012) or mental disorder, such as schizophrenia (Boyer et al., 2012; Cohen et al., 2022; Hsiao et al., 2020; Khatimah et al., 2022; Margetic et al., 2013; Meng et al., 2021; Yerriah et al., 2022). The generalizability of these findings across different countries and conditions confirms that, despite cultural variability, healthcare system differences, and variations in symptoms and disease presentation (Beentjes et al., 2012; Broese van Groenou and de Boer, 2016; Verbakel, 2018; Zendjidjian et al., 2012), there is a degree of commonality in the caregiving experience that is reflected in the caregiver quality of life.

Our study holds significance as one of the few conducted on the quality of life of informal caregivers for persons with mental health problems in the Netherlands. Other quality of life research conducted in the Netherlands found similarly low quality of life scores in this caregiving population (Hastrup et al., 2011). Hence, informal caregivers for persons with mental health problems constitute a particularly vulnerable group of caregivers in the Netherlands (de Boer et al., 2019). With the growing burden of mental disorders and the significant increase in reliance on informal care in the Dutch healthcare context (Broese van Groenou and Boer, 2016; McGrath et al., 2020; Walker et al., 2015), the well-being of informal caregivers for persons with mental disorders is an increasingly important topic. Several initiatives have been taken at the national (e.g., the introduction of the Flexible Employment Act in 2016) and local level (e.g., provision of temporary residential care) to support the general informal caregiver population; however, relatively few informal caregivers make use of these services. Several factors may contribute to this, including insufficient or inaccessible information and reluctance of informal carers to seek support (De Boer et al., 2020b). Stigma may also contribute to this phenomenon, in the case of mental health disorders. Informal caregivers of persons with mental health problems often feel lonely and isolated due to the nature of mental disorders and disorder related-stigma (Greenwood et al., 2018; Hayes et al., 2015). Additionally, this population of caregivers is often excluded from their care recipients’ treatment process (Seif et al., 2022) and subjected to courtesy stigma (Angermeyer et al., 2003).

We identified several modifiable determinants associated with a low quality of life, including subjective burden, psychological well-being, and social support network. These determinants are widely recognized in caregiver quality of life research (Caqueo-Urízar et al., 2009; Cheng et al., 2022; Cohen et al., 2022; Gräler et al., 2022; Jorge et al., 2019; Khatimah et al., 2022; Richieri et al., 2011). Empirical literature suggests that addressing these modifiable determinants with positive coping skills can enhance informal caregivers’ subjective appraisal of their informal care provision. Crucially, the caregiver’s appraisal of their informal care provision is causally linked to the negative consequences of caregiving and predicts whether negative outcomes, such as a poor quality of life, will be experienced (Gräel and Adabbo, 2011). The Transactional Model of Stress and Coping by Lazarus and Folkman (1984) illustrates how negative caregiving outcomes result from the informal caregiver’s perceived discrepancy between the appraisal of a distressing caregiving situation and their ability to cope (Gräel and Adabbo, 2011; Lazarus, 1966; Lazarus and Folkman, 1984).

We also identified several non-modifiable risk factors that were associated with caregiver quality of life. The effects of caregiver age (Hsiao et al., 2020; Wong et al., 2012) have been mixed, whilst the role of child caregiving responsibility (Di Novi et al., 2015) has received limited attention in the quality of life literature. We found a protective effect of old age, which could be due to older caregivers becoming more adjusted to their caregiving role and acquiring additional skills in managing their care recipient’s condition over time (Wong et al., 2012). This protective effect can be explained by the *Adaption Hypothesis*, whereby informal caregivers develop coping skills over the duration of caregiving and derive new purpose from their caregiving situation (Townsend et al., 1989). We found that child caregiving responsibility was associated with a lower caregiver quality of life. This has also been evidenced in a quality of life study focused on female informal caregivers. Additional responsibilities, such as childcare, require informal caregivers to have a better balance between their informal care provision and other responsibilities and hence, child caregiving may have a significant influence on informal caregivers’ quality of life (Di Novi et al., 2015). Our inclusion of this variable may also explain why we did not find a significant impact of female gender on quality of life. Gender is generally accepted as an important quality of life predictor, despite inconsistent documentation for informal caregivers of persons with mental disorders (Sharma et al., 2016).

### Implications for future research and healthcare policy

This research underscores the importance of developing targeted caregiver interventions and policies, particularly tailored to the unique challenges of caring for individuals with mental disorders. To address this, future research should focus on evaluating the effectiveness of various forms of caregiver support in meeting the needs of caregivers in the Netherlands (De Boer et al., 2020b), especially those who care for individuals with mental disorders. These interventions should take into account caregivers’ subjective appraisals of their caregiving experiences, aiming to enhance their quality of life by promoting positive coping strategies. Moreover, it is crucial for these interventions to incorporate opportunities for caregivers to take breaks from their caregiving responsibilities, allowing them time for personal pursuits and other obligations (Di Novi et al., 2015; Jeon et al., 2005). However, it is essential for these services to be flexible, recognizing the unpredictable nature of mental health challenges and accommodating the specific needs of this caregiving demographic (Jardim and Pakenham, 2010; Jeon et al., 2005). By doing so, caregiver interventions can better support individuals providing informal care while also considering the nuanced demands of mental health caregiving.

Additionally, future quality of life research in informal caregivers for persons with mental health problems should prioritize the adoption of longitudinal research methodologies. Longitudinal studies provide a robust framework for establishing causality and allow researchers to assess changes in caregiver quality of life over time. By assessing informal caregivers’ experiences and outcomes across multiple time points, insight can be gained into temporal patterns and any potential mediating or moderating factors influencing other relationship between the determinants and caregiver quality of life (Rothman and Greenland, 1998). Some research has already been conducted in a population of Taiwanese caregivers for patients with schizophrenia, major depressive disorder and bipolar disorder on the mediating effect of self-esteem and psychological distress on the relationship between caregiving burden and overall quality of life using an adapted version of the Stress Process Model. They found that caregivers’ psychological health and caregiving burden significantly influenced informal caregivers’ overall quality of life. However, this study utilized a cross-sectional dataset and has a low external validity. Hence, more research is needed to further investigate these relationships (Cheng et al., 2022).

### Strengths and limitations

This is a study that investigated the quality of life of informal caregivers for persons with mental health problems and identified relevant determinants by employing a caregiver specific-quality of life instrument, the CarerQoL (Brouwer et al., 2006). The utilization of this instrument allowed us to assess caregiver quality life-a distinct construct that is separate from overall quality of life. The former exists within the specific context of informal care provision, whilst the latter can be attributed to any life situation, role, or issue. Hence, an informal caregiver may report feeling satisfied with their life and also experience issues related to their informal care (Martin et al., 2021).

Although this study provides an initial insight into caregiver quality of life and its respective determinants, further research is needed to better understand the role of additional factors, such as the diagnosis, additional sources of caregiver support, and coping skills (Sonido et al., 2022; Van Wijngaarden et al., 2009; Wong et al., 2012) and the possible moderating and mediating role that these factors may have (e.g., Cheng et al., 2022). We also recommend validating the results using longitudinal research to capture changes in quality of life over time and explore causal relationships.

There are also additional limitations that should be addressed. First, our results may represent an overestimation of caregiver quality of life in this study population due to sampling bias. Informal caregivers who have an exceptionally low quality of life may not have had the motivation or resources to participate in the panel study survey.

Second, data collection occurred during the first wave of the COVID-19 pandemic in the Netherlands. Significant reforms were implemented in the Dutch (mental) healthcare system at this time, with the partial suspension of services (e.g., Dinmohamed et al., 2020; Van Manen et al., 2021) and rapid implementation of telemedicine (Chow et al., 2021; Feijt et al., 2020). These factors may have had an impact on the quality of life of informal caregivers (Gräler et al., 2022; Zwar et al., 2023). Notably, other aspects of COVID-19 pandemic may have also impacted informal caregivers’ quality of life, such as the high degree of uncertainty (De Boer et al., 2020a) and the economic turmoil present at this time, as evidenced in other study populations (Brooks et al., 2020). To a certain extent, we controlled for this effect in our analysis with inclusion of the control variable, COVID-19 related concerns.

Third, when compared to a representative panel of Dutch informal caregivers (Netherlands Institute for Social Research, 2020) our study population was largely comparable, with the exception of higher unemployment rates and longer caregiving duration. It is unclear what the implications of this could be on the quality of life scores in this study population. Given that there are mixed results concerning the benefits of employment on caregiver quality of life. However, employment can aid in ensuring the informal caregiver’s economic well-being, prevents professional activity decrease and earning loss, and could cover additional informal care expenses (e.g., patient treatment costs; Simon, 2003). Longer caregiving duration may have a protective effect on quality of life (Leng et al., 2019; Nogueira et al., 2019). The protective effect of a longer duration of caregiving may be explained by the *Adaption Hypothesis* (Townsend et al., 1989).

## Conclusion

In conclusion, this research has unveiled a profound reality concerning the quality of life for informal caregivers of individuals with mental health problems in the Netherlands, confirming an exceptionally low level of well-being within this caregiving population. The identified determinants—subjective burden, perseverance time, caregiver overall health status and psychological well-being, caregiver age, child caregiving responsibilities, and social support network—underscore the multifaceted nature of the challenges faced by these caregivers. In light of these findings, it is imperative for policymakers, healthcare professionals, and support organizations to develop comprehensive and targeted interventions. These interventions should address the identified determinants, acknowledging the dynamic and interconnected nature of the challenges faced by informal caregivers.
